# Experimental investigation of the mitigation of bubble collapse loads due to the free surface^☆^

**DOI:** 10.1016/j.ultsonch.2025.107459

**Published:** 2025-07-07

**Authors:** Rho-Taek Jung, Nyo Me Thet Naing, Dong Kim

**Affiliations:** a Foundation for Industry Cooperation, University of Ulsan, 93 Daehakro, Nam-gu, Ulsan 44610, South Korea; b School of Naval Architecture and Ocean Engineering, University of Ulsan, 93 Daehakro, Nam-gu, Ulsan 44610, South Korea; c School of Mechanical Engineering, University of Ulsan, 93 Daehakro, Nam-gu, Ulsan 44610, South Korea

**Keywords:** Low-voltage spark generation, Polyvinylidene fluoride, Impulsive forces, Free surface, Air-pocket

## Abstract

Small bubbles were generated beneath the free surface using the electric spark method. Their behavior was observed through high-speed photography, and the impact forces were measured using a polyvinylidene fluoride (PVDF) sensor attached to the vertical solid wall and horizontal wall above an air pocket in a groove plate. The proximity parameter γ_w_ and γ_f_ were defined as the non-dimensionalization for the free surface and vertical wall, respectively, and two-dimensional map of the impact load and jet orientation was presented based on the parameters. The findings indicate that the position of the bubble center, where the maximum force is generated as it approaches the free surface, is located about two times farther from the vertical wall compared to the region where the free surface has no influence. Additionally, the jet direction is directed toward the lower side of the water surface and perpendicular to the wall. Moreover, the impact force on the wall was also measured when the air gap existed between the bubble and the wall. In this case, the measured force was significantly smaller compared to when no air gap was present. The experimental data suggests that the presence of an air gap can effectively reduce the impact force generated by the bubble which smaller than the air gap size.

## 1 Introduction

Once the vapor-filled bubble forms in a quiescent fluid environment, it undergoes expansion until it reaches its maximum size. Following this, the bubble begins to contract, collapse, and rebound, generating a high-velocity jet and shockwaves that propagate toward the boundaries over a very short time scale. The collapse of cavitation bubbles generates intense forces and energies that can lead to material damage. In sonochemical engineering, controlled bubble collapse facilitates high-intensity reactions, essential for applications like organic synthesis and nanomaterial fabrication [1,2]. Cavitation bubbles, formed by ultrasonic waves, create extreme local temperatures and pressures that drive efficient chemical processes [3]. Cavitation is particularly concerning in ocean engineering, where bubble-induced cavitation can cause severe damage to marine propellers and turbine blades, hindering performance and durability [[4], [5], [6], [7], [8]]. In medical science, bubble cavitation plays a crucial role in drug delivery and cancer treatment through processes like ultrasound-mediated therapy. Bubbles are used to enhance the penetration of drugs into tissues, but unwanted collisions or unstable cavitation could cause tissue damage or hemorrhage. [9,10]. Therefore, precise control of bubble dynamics is critical to avoid detrimental effects [11,12]. In food science, cavitation is employed for processes like emulsification and homogenization. While it facilitates the breakdown of particles into finer sizes, excessive bubble collisions could lead to over-processing and altered product properties [[13], [14], [15]].

In the cavitation study, the phenomenon itself is very complicated by the impact load caused by the collapse of the surrounding mutual bubbles and the shock wave caused by the collapse of a large amount of bubbles through the repetitive process of expansion and contraction, and if shear flow is generated to it like a propeller, it can be a very complex phenomenon. Therefore, due to the complexity of this cavitation, it needs to be simplified into hydrodynamic cavitation of a single bubble. Therefore, the three typical methods of generating a single bubble are as follows.

Laser-induced cavitation is ideal for precise, small-scale experiments with precisely controlled bubble sizes, focal point, and timing. A high-energy laser pulse (such as a Nd:YAG laser) is focused onto the water, causing rapid heating at a localized spot. This intense heating vaporizes the surrounding water, generating a vapor bubble that rapidly expands and then collapses, generating a shock wave [[16], [17], [18], [19], [20]]. Spark-induced cavitation offers a flexible and cost-effective method for generating bubbles of varying intensity. A high or low voltage electrical spark is discharged between two submerged electrodes. The energy from the spark heats the surrounding water, causing rapid vaporization and bubble formation. It’s relatively easy to set up, requiring just electrodes and a spark generator. By controlling the voltage, we can vary the energy of the spark and, consequently, the size and intensity of the bubble [21,22]. Detonator-based methods are best suited for large-scale experiments and realistic explosion studies, but are impractical for small-scale experiments due to safety concerns, lack of control over bubble size, and high energy consumption. A small explosive charge (such as TNT) is placed underwater [[23], [24], [25]].

The stand-off parameter, defined as the ratio of the distance from the point of inception to the wall over the bubble radius, is widely used in bubble cavitation research. When a single bubble collapses very close to a solid wall, it is expected to initiate with a very high pressure, which subsequently decreases as the distance from the wall increases. However, experiments have shown that there exists a range in which the pressure during the first collapse is locally diminished due to the splashing effect when the bubble collapses slightly away from the wall, specifically at stand-off values between approximately 0.8 and 1.2. Additionally, the pressure associated with the second collapse is generally lower, as most of the energy is dissipated near the wall during the first collapse. Nevertheless, experimental results confirmed that the second impact exhibits a pressure value comparable to the maximum pressure of the first impact at the point where the splashing effect from the first collapse ceases [26,27].

For clarity, [28] utilized a high-speed camera operating at 100 million frames per second to observe the bubble dynamics. The maximum bubble growth reached a radius of 1.5 mm, and they carefully concerned the toroidal-like bubble, the formation of the splash, and the counterjet, all observed at a 45-degree angle from the wall and analyzed that the major torus radius increased slightly at γ = 0.8, while it decreased at γ = 0.3. This suggests that the initial development of the major torus radius before the subsequent spreading process plays a crucial role in minimizing impact load at the stand-off range of approximately 0.8 to 1.2.

When a bubble collapses near a convex solid boundary, especially for wall curvature, the jet velocity is much higher than that of the flat boundary case, calculated by the boundary integral formulation theoretically. It is noted that the jet velocity calculated is two times faster as the stand-off parameter changes 1.0 to 0.5 [29].

To assess the impact pressure loading on both the solid plate wall boundary and the hemispherical boundary, a Hopkinson bar apparatus is employed. A 5-mm diameter sensor is utilized to measure the collapse load of a maximum 60-mm diameter bubble [30,31]. The pressure at the hemispherical boundary reaches 40 MPa, while at the plate boundary, it is 10 MPa, with a stand-off parameter of 0.67. The pressure profile at the hemispherical boundary exhibits no localized low-pressure values, in contrast to the plate boundary. Additionally, the second collision-induced pressure peaks at the hemispherical boundary are more pronounced than the first, a pattern also observed at the plate boundary. The PCB Piezotronics is employed to quantify pressure magnitude, while hydrophones are commonly utilized to measure underwater shock mechanisms [22,32,33]. Alternatively, a piezoelectric polyvinylidene fluoride (PVDF) polymer film, approximately 28 μm in thickness, is also effective in measuring impulsive forces applied to the wall. The high sensitivity, excellent durability, and broad bandwidth of the PVDF sensor make it ideal for high-amplitude impulsive field measurements [34,35].

The dynamics of bubble cavitation involve multiple phases, including generation, expansion, collapse, and rebounding, which occur repeatedly. The first and second impacts are the most dominant factors influencing this process. Investigating the direction and velocity of the bubble jet is a critical aspect in understanding the nonspherical collapse of bubbles. Since the early work of [36], it has been observed that bubble collapse near a flat solid boundary results in a jet directed toward the boundary. The formation of this jet suggests that a portion of the impact area is concentrated on the boundary. In complex geometries, such as rigid perpendicular walls or patterned surfaces, [37,38] experimentally examined bubble collapse and jet formation at corners formed by two flat solid boundaries with varying opening angles. When a bubble is generated equidistant from the walls, the resulting jet is directed along their bisection. The motion of the jet is highly sensitive to the stand-off distance from the rigid wall [39].

In the presence of a free surface, a thin jet within the bubble and a re-entrant jet extending away from the free surface can be observed through high-speed imaging [40]. During the initial growth phase up to the first collapse, a water spike is generated, accompanied by the contraction of the re-entrant jet and the formation of a toroidal bubble. The jet torus moves downward as the first collapse occurs, and the shockwave generated during this collapse induces an inertial water skirt on the free surface. The vertical length of both the spike and skirt increases until the inertial effects subside. [41,42]. Since the maximum bubble size of these experiments are about 1/3 of the water depth, it can be seen that the energy dissipation through the spike on the free surface is maximized, and the complex propagation and reflection of the shock wave were well captured through the Schlieren method.

Single bubble research extends to complex surrounding boundaries experimentally and numerically, such as vertical walls and free surfaces [43], air bubble attached to wall boundary [44], gas-entrapped interfaces [45,46], and elastic materials [16] (using polyacrylamide(PAA) with various water contents and [47] for Silicons and Aluminium. In this context, [45] and [46] investigated the behavior of entrapped gas in Gas-Entrapping Microtextured Surfaces (GEMS), which utilize hydrophobic coatings to trap air in cavitation bubbles within a stand-off range of 0.7 to 5.1. The maximum radius of the bubble at a stand-off parameter of 5.1 is 0.61 mm, while the microtextured surface has a diameter of 0.2 mm and a depth of 0.05 mm. At a stand-off parameter of 5.1, the affected texture region extends to a diameter of approximately 1.8 mm, which is slightly larger than the maximum bubble diameter. Notably, after the first collapse, the bubble jet is repelled and may not undergo a second collapse due to the energy absorbed by the entrapped air, a behavior that dominates in this scenario. The trapped gas or air pockets can be effectively utilized in surface engineering to reduce the bubble impact force on solid surfaces. This approach can help minimize cavitation noise in underwater propellers and fluid machinery [48], and can also be applied in biomimetic GREMS structures on SiO_2_/Si surfaces, as previously discussed [45,46].

Tian et al. [49] successfully simulated that peak pressure impulse on the biomimetic GEMS is reduced by 33 % effectively mitigating the degree of damage to the wall using the compressible multiphase VOF(Volume of Fluid) model. Also [43] investigated Interaction between a nonspherical pulsating bubble and a free surface near a solid wall with the Eulerian finite element method and the adaptive mesh refinement technology that realize the nonlinear interaction between a bubble and a free surface.

The impact load resulting from the bubble's collision with the wall was measured using a PVDF sensor mounted on the wall surface. By varying the bubble generation position, a two-dimensional impact load distribution map was constructed. While many previous studies [22,32,33] have used hydrophone-type sensors to measure impact loads, this study utilized PVDF sensors to specifically quantify the load transmitted to solid boundaries. In cases involving mixed boundaries (combinations of solid walls and free surfaces) accurate measurement of impact loads remains challenging. This complexity was further addressed through an analysis of the impact load distribution near corners, where the presence of a free surface significantly influences the measured values. Additionally, an experiment was conducted to evaluate the impact load transmitted through a thin air layer. The results demonstrated that the bubble-induced impact load is significantly attenuated by the presence of a confined air layer within grooved regions. Further studies are required to investigate the influence of air layer thickness and groove geometry on the transmission characteristics of impact loads.

This paper is dedicated to measure the impulsive load investigating bubble collapse behaviors near both free surfaces and solid boundaries, as well as the corresponding impact loads on the wall boundary passing through an air pocket. In Section 3, the interactions between a vertical wall and free surface are examined in a laboratory-scale experiments. Section 4 investigates the behavior when both the vertical wall and free surface are in close proximity. Section 5 discusses the impact measured by a PVDF sensor attached to a grooved plate, located beneath the air pocket, as a bubble collapses within this configuration. Finally, Section 6 provides a summary and conclusion.

## 2 Experimental setup

**Fig. 1:**
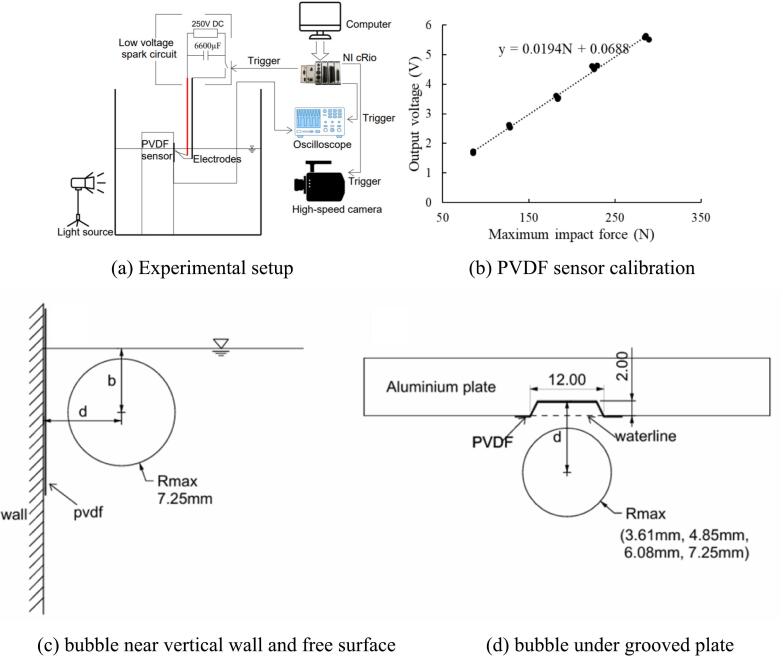
Experimental preparation.

In this study, the water tank of 400 mm × 400 mm × 450 mm is used and filled with tap water at room temperature, see Fig. 1(a). The tinned copper wire electrodes (0.16 mm in diameter), which are in contact initially, are connected to a low-voltage discharge circuit. Since the maximum bubble radius induced by this configuration is between 3 to 16 mm (by adjusting the discharge voltage), interference from thin electrodes, which are more than 30 times smaller than the bubble, is neglected. The spark-discharge circuit is inspired by[50]. This study uses the N-channel metal–oxide–semiconductor field effect transistor (MOSFET) (IXYS IXFH220N20) to discharge the energy stored in capacitors. The three capacitors of 2200µF 250 V are connected in parallel, thus giving a total capacitance of 6600µF. The backlight source (200 W) is used for illuminating the high-speed camera through a translucent acrylic sheet. Images are recorded with a Chronos 1.4 high-speed camera. A thin film (28 µm) of PVDF piezoelectric transducer (FDT1-028 K) is attached to the boundary near the spark to quantify the impulse signals produced by cavitation bubble collapse. The sensor is connected directly to the oscilloscope (PICOSCOPE 5443D, 100 MHz, maximum sampling rate 1GS/s). NI LabView Graphical User Interface is employed to interact with NI cRIO-9047. The cRIO sends synchronous trigger signals to a spark circuit, high-speed camera, and oscilloscope.

When a bubble collapses near a solid surface, it generates an impact on the wall. This impact can cause the bubble to rebound into a toroidal (donut-like) shape or form satellite bubbles, leading to a secondary collapse. There are two key considerations when sensing bubble impacts on solid surfaces. First, the sensing area must be sufficiently large. The sensor must capture both the primary and secondary impacts. Since satellite bubbles can cause a wider secondary impact area than the initial collapse, the sensor’s active area must be large enough to account for this. This requirement increases the cost of the sensor. Second, the sensor must conform to the geometry of the solid wall. It should integrate seamlessly with the surface, without introducing discontinuities. The mounting method of conventional (transitional) transducers must ensure a precise fit to maintain measurement accuracy. In this study, a PVDF film sensor was used to measure bubble impacts. PVDF is relatively easy to install and maintains good conformity with complex wall geometries. For comparison, we also examined data from a traditional sensor (Swiss Kistler 603B, 5.55 mm diameter, 400 kHz resonant frequency) reported by Tomita and Shima [26]. The PVDF sensor showed a similar trend in maximum impact pressure as a function of the γ value. More detailed analysis is available in our previous work [35,51].

The PVDF (polyvinylidene fluoride) film sensor features a sensing area of 12 mm × 30 mm and is fabricated with elongated silver ink electrodes printed on both surfaces of a die-cut polymer substrate with a thickness of 28 µm. Signal output is facilitated via connector pins attached to the distal ends of 100-mm-long silver electrodes. According to the manufacturer, the nominal sensitivity of the sensor is 0.013 V/N. In this study, the sensor is encapsulated with a 50-µm-thick protective film for waterproofing and mechanical protection, which may alter the sensitivity depending on the thickness of the protective layer and the nature of the mounting surface. The impact testing is conducted using the ball drop method, in which stainless steel balls are released from a height of 500 mm through an acrylic guide tube. The mean impact force is calculated using the impulse–momentum theorem as formulated by Hujer and Müller [52]. As illustrated in Fig. 1(b), the measured sensitivity is 0.0194 V/N, which exceeds the nominal value provided by the manufacturer. This increase is attributed to the additional protective layer. Furthermore, the output voltage exhibits a strong linear correlation with the applied impact force. Dynamic performance testing in a gas dynamic shock tube, as reported by Wang and Chen [34], shows that the PVDF sensor demonstrates a rise time of 56 ns and a flat frequency response in the range of 0 to 1 MHz.

The bubble is created in close proximity to both free surface and a vertical wall, as shown in Fig. 1(c). The proximity parameters for the wall and free surface are γ_w_ = d/Rmax and γ_f_ = b/Rmax. The maximum bubble radius is 7.25 mm at 82 V discharged voltage (in free field measurement), and the normalized ranges are γ_w_ < 5.0 and γ_f_ < 6.0. It should be noted that the vertical wall is fixed at the same position while the water level is adjusted for desired stand-off parameters. In Fig. 1(d), the air gap is maintained(2 ∼ 3 mm) under the grooved plate, and the bubble is incepted in the range of γ < 4.0. Four bubble radius sizes (3.00 mm, 4.85 mm, 6.08 mm, and 7.25 mm) are created to analyze the influence of air gap on bubble dynamic behaviors. Another parameter, λ = 2Rmax/12, is introduced for this case (where 12 is the groove length in mm). The cushion effect of an air pocket when the bubble collapses is investigated.

To minimize positional inaccuracies in the spark-induced method, each experiment was conducted in minimum triplicate. This repetition mitigates inconsistencies or variations in electrode alignment, thereby enhancing the precision and reliability of the results. The bubble radius in this study ranged from 3.61 mm to 7.25 mm. Four different applied voltages, ranging from 50.0 V to 81.5 V, were used to control bubble generation. To determine the maximum bubble radius corresponding to each applied voltage, measurements were conducted four times for each condition. The average value maintaining a standard deviation within 5 % was adopted as the representative maximum radius, as summarized in Table 1. The results indicate that the bubble size increases approximately linearly with increasing applied voltage.

**Table 1:** Bubble size setting thru voltage.

voltage	radi1	radi2	radi3	radi4	Avg.	Std.
50.0	3.62	3.61	3.63	3.58	3.61	0.017
60.5	4.80	4.85	4.87	4.86	4.85	0.027
69.5	6.05	6.06	6.10	6.09	6.08	0.021
81.5	7.23	7.20	7.23	7.34	7.25	0.053

## 3 Bubble near a single boundary

### 3.1 Bubble collapse near the free surface

**Fig. 2:**
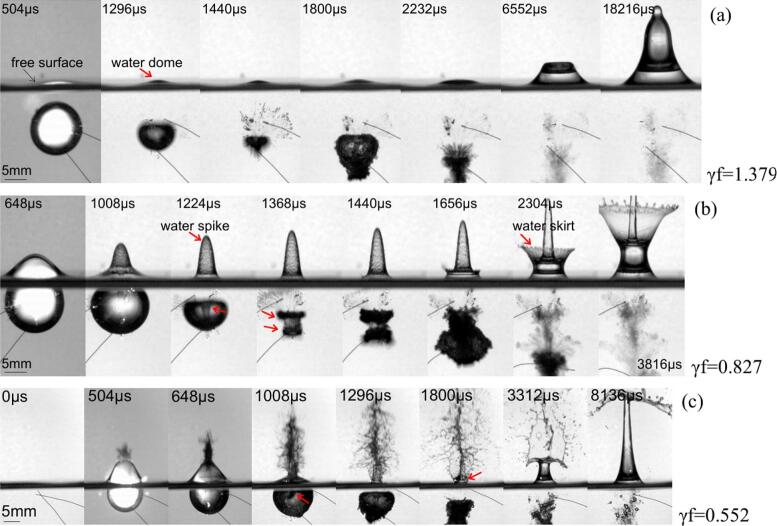
Bubble shape evolution under the free surface.

In this section, the bubble is generated under the free surface and is placed away from the vertical wall. The growth and collapse phases of the bubble are weakly affected by the free surface for the stand-off distance (γ_f_ = 1.379), see Fig. 2(a). The outward flow from expanding bubble pushes the free surface above it to form the water dome at the early stage, and the top bubble surface is slightly elongated. In other words, the flow velocity is higher in the region between the free surface and the bubble than in other parts of the bubble, and neighboring water along the still water line is drawn to the dome formation. When the bubble enters the collapse stage, two flow directions form above the cavity, upward flow to the dome and downward flow to the shrinking cavity[53]. This results in the formation of a high-pressure stagnation region along the axis of symmetry. The water dome is relatively small for this proximity parameter, hence low flow intensity and weak inertia. Thus, upward flow is attenuated, and the water dome falls back to still water level by strongly attaching to the collapsing bubble. Meanwhile, the top bubble wall is flattened, and jet formation is not clearly visible before reaching the minimum volume. During re-expansion, the bubble maintains the flat top profile, and the funnel through the bubble interior is developed at the bottom part. Eventually, it is repelled by the free surface and migrates downward after several oscillations. This pulsating bubble also manifests the cone-shaped structure above the waterline after approximately 13 collapse periods(18 μs).

Similar collapse behavior is observed for smaller proximity parameter (γ_f_ = 0.828) from the boundary, see Fig. 2(b). In this case, the bubble is closer to the free surface, and its top surface is entrained underneath the raised free surface. Subsequently, a higher water dome is produced by bubble enlargement. The dome's base is the broadest at the maximum bubble volume but falls back upon bubble collapse. Afterward, the dome transforms into a water spike along the symmetry axis. Analogous to the previous case, upward fluid flow to the spike, and downward fluid to the contracting cavity, forming a high-pressure region above it. The wide liquid jet is distinguishable even at the beginning of the collapse phase; see a red arrow at 1,224 µs in the corresponding photograph panel. The involution of the bubble reveals a flat top bubble surface and forms the toroidal bubble. In literature, this collapse dynamic is investigated by a 10 million fps high-speed camera and demonstrates the shock wave emission at the jet impact, and the toroidal bubble collapses [54]. Upon jet impact, the crown splash appears at the spike root part on the free surface, liquid propagating along with the bubble interface. The main bubble contracts, and recirculated liquid propagates downwards along the main jet. The main bubble collapse first, and the jet-induced torus collapse later. The outward flow of the rebound bubble interacts with the water spike event and uplifts a relatively large liquid bulk underneath the spike. This effect develops the water skirt and becomes more and more pronounced. The pulsating bubble migrates far from the free surface, but the spike and skirt continue to rise under the inertia.

The bubble exhibits more violent collapse deformation when it is created very close to the free surface (γ_f_ = 0.552), see Fig. 2(c). The dome formation is faster and has a broader base for this small distance. The top bubble surface is extremely stretched and breaks the surface tension. This observation is also reported in the literature [55]. The bubble bursts into the open air, resulting in pressure changes inside the bubble. This situation forces the bubble to shrink even before achieving the maximum volume. Accordingly, the collapse period is shorter near the free surface. The breaking water layer becomes a very unstable water spike, and high-speed jet penetration inside the bubble is recorded. Bubble develops a very rough interface, and liquid jet impacts on the opposite wall. The bubble is presumably normalized to the atmospheric pressure. After that, the water skirt develops and elevates to a high distance above the still water level.

When a bubble explodes under a free surface, its behavior changes depending on how close it is to that surface. If the bubble is very near the free surface, the collapse generates a jet directed upwards, often resembling a spike. This happens because the fluid is forced upwards by the compression caused by the bubble’s collapse and the presence of the surface, which restricts the bubble’s expansion. The collapse is more concentrated in the upward direction due to the fluid’s confinement. However, if the bubble is a bit farther from the free surface, the jet formation changes. The collapse of the bubble, while still asymmetric, is no longer focused upward. Instead, the fluid is directed away from the free surface, pushing in the opposite direction. The fluid's compression and the bubble’s collapse dynamics cause the energy to be displaced downward or to the sides, creating a jet that moves opposite to the surface.

### 3.2 Bubble collapse on a flat rigid boundary

In an unbounded fluid domain, the pressure on the bubble surface remains uniformly distributed during growth and collapse. However, in the presence of nearby boundaries, such as a solid wall or free surface, the bubble surface pressure becomes non-uniform. This asymmetry induces a jet directed from the low-pressure region toward the high-pressure side.

When a solid wall is present near the bubble, the initially spherical symmetry of the bubble is disrupted. As the jet penetrates through the bubble's center, it evolves into a high-speed microjet that first impacts the solid wall. Subsequent impacts are generated due to the toroidal-shaped bubble's collapse and re-expansion phases. The magnitude of these impact loads depends on the stand-off parameter (γ), which denotes the normalized distance between the bubble center and the wall.

**Fig. 3:**
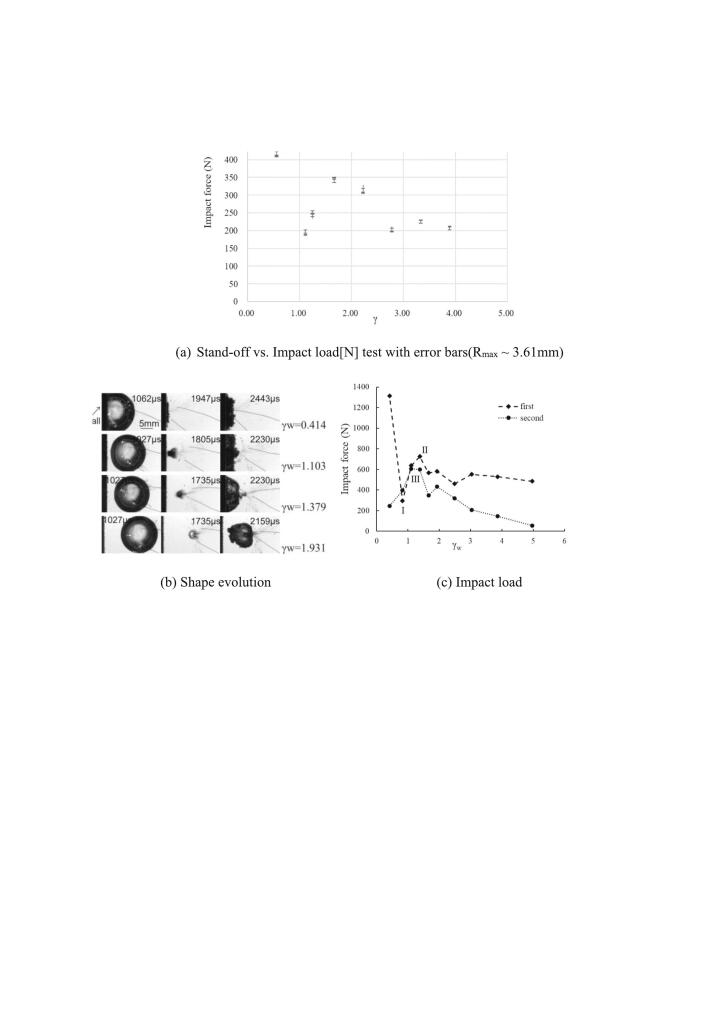
Single bubble collapses on the flat boundary as a function of γ_w._

When conducting bubble impact experiments using the low-voltage spark method, it is essential to evaluate potential experimental errors. These may arise from electrical, mechanical, or human-related sources. To minimize such errors, the experiments were carefully repeated multiple times under identical conditions. Fig. 3(a) presents the results of the measured impact load [N] as a function of the stand-off parameter, with each condition tested three times. Corresponding error bars are included to indicate measurement variability. A distinct concentration region referred to as the “hump zone” is observed near γ ∼ 1.

The magnitude of impact force on the solid boundary varies with the proximity parameter. Fig. 3(b) shows the maximum impulsive forces generated from the first and second collapses according to their corresponding proximity parameters. After the first collapse, the toroidal tiny bubble cloud approaches to the vertical wall while staying away from the case of the free surface in the previous section. In this section, the bubble is placed 20 R_max_ under the still water level, which would keep it completely isolated from the free surface. In Fig. 3(c), we can see from the graph that smaller γ_w_ gives the larger force reading for the first collapse. The closer to the boundary, the more destructive the bubble becomes. On the other hand, second collapse forces are getting more pronounced in the interval (1 < γ_w_ < 2). Interestingly, when the distance, d, is approaching the maximum radius (γ_w_ ∼ 0.8), the first impact force becomes less destructive compared to the larger value. This is in accord with the experimental findings of [26,34,56] and [51]. In their studies, the small valley in pressure output of the first collapse is evident for 0.6 < γ_w_ < 1.1. Although there are fluctuations in peak values for γ_w_ > 2, the first collapse impacts are always higher than the second one. The force trends show a decreasing pattern with increasing γ_w_.

The γ_w_ range happening the valley zone, the bubble is positioned at the closed distance from the wall. At this distance, the energy is not as efficiently transferred to the wall because the compressed water layer acts as a buffer, and the flattening of the proximal prevents the bubble from focusing its collapse directly onto the wall in the most forceful manner. Because the bubble's proximal is flattened and the compressed water layer exists, the dynamics of the shock wave and bubble collapse are affected by fluid resistance and the viscous forces in the thin water layer. This layer cushions the shock wave and helps dissipate the energy. When the stand-off ratio is larger (γ_w_ > 1.2), the compressed water layer effect on the proximal becomes less significant because the bubble is farther from the wall. The shock wave retains more of its energy as it travels to the wall, and there is less flattening of the proximal of the bubble. This leads to a more concentrated collapse and a stronger impact when it reaches the wall, which results in a larger measured force.

The initial collapse of the bubble typically occurs rapidly and is often associated with the highest energy release. The shock wave produced by this first collapse is strong, creating a high-intensity impact load on nearby surfaces. However, the energy released in this first collapse can be somewhat dissipated by the surrounding fluid, and the shock wave loses some of its focus after the initial impact. After the first collapse, the bubble tends to rebound slightly before collapsing again. These subsequent collapses are often less intense than the first but can still produce significant pressure waves. Importantly, the second collapse can often generate a higher impact load than the first, especially at certain proximities to solid surfaces.

## 4 Bubble near the mixed boundaries between free surface and vertical wall

The asymmetric or uneven bubble collapse behavior is prevalent when the bubble is subjected to the combined actions of two boundaries system. When a bubble explodes in an environment where both a free surface and a wall are present, the collapse dynamics become more complex [43]. The free surface tends to push the fluid upward when the bubble is near it (especially very close).

**Fig. 4:**
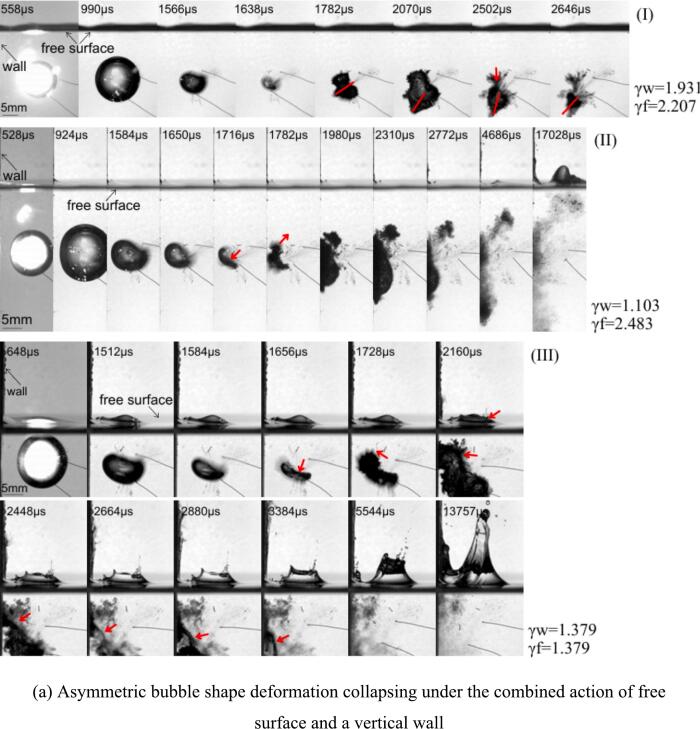

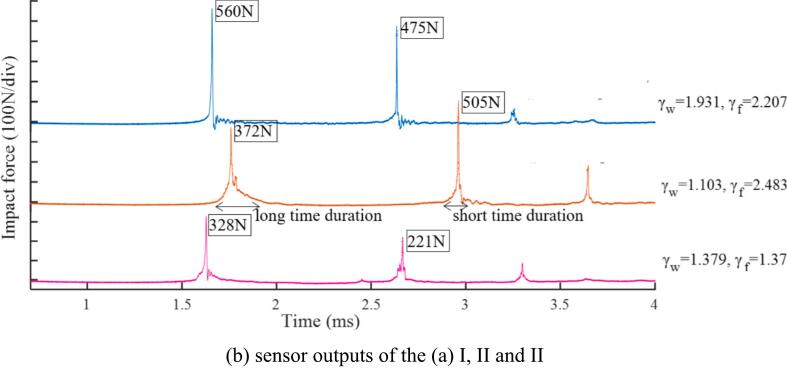
Typical bubble collapse vicinity both free surface and solid wall.

However, as the bubble moves farther from the free surface, the collapse no longer results in a purely upward jet. Instead, the collapse redirects the fluid due to the proximity of the second boundary (the wall). When the bubble is close to the wall, the fluid is funneled towards the wall, creating a horizontal or slightly angled jet directed towards the solid surface. In this experiments, the distal bubble part (away from the nearby boundary) collapses first, and this compressive wave interferes with the proximal part's collapse process. The changes in the jet direction inside the second rebound bubble are evident for the bubble incepted at γ_w_ = 1.931 and γ_f_ = 2.207; see case I of Fig. 4(a). The rebound of the distal part has a larger volume than that of the proximal portion.

During re-expansion, the funnel shape at the bubble bottom shifts its orientation. Subsequently, two parts merge, and the proximal part starts to collapse first in the second collapse phase. The first and second impact peaks are 560 N and 475 N, respectively. In case II of Fig. 4(a), the bubble is created with γ_w_ = 1.103 and γ_f_ = 2.483; thus, the wall influence is expected to be greater than the free surface. However, the pronounced inclined jet is induced by the free surface effect at the first collapse stage. As a result, the asymmetric collapse occurs, and the rebounds of two bubble parts expand in opposite directions, as evident in the pictures. It can be postulated that the collapse load from the distal part hits the boundary and reflects in the jet direction. This interacts with the proximal part and repels it away from the boundary. The first collapse load is lower than the second (370 N and 500 N) for this particular γ_w_ value which is the same manner reported in the previous flat rigid boundary section. When the bubble is located equal distance from both boundaries (γ_w_ = γ_f_ = 1.379); thus, interesting bubble evolution is captured, see case III of the Fig. 4(a). The typical inclined jet, asymmetric collapse, and rebounds are similar to the general case until the proximal rebound touches the wall. Soon after the contact, the bubble enters the second collapse phase. Due to boundary retardation, bubble parts near the boundary shrink insensibly while the opposite parts contract faster, forming a pinch-off-like collapse (1,656 μs (III) in Fig. 4(a)). Eventually, the original bubble is segregated into two main parts, but they are still connected via a thin channel (due to the second collapse 2448 μs ∼3384 μs (III) in Fig. 4(a)) marked with an arrow in the photograph. Although the bigger part migrates downward faster than the smaller part (attached to the wall), the channel link is not broken, and they oscillate for several cycles afterward.

Fig. 4(b) displays the impact load outputs corresponding to the cases shown in Fig. 4(a) I, II, and III, respectively. The recorded values for the first, second, and third impacts are clearly distinguishable. In case I, where γ_w_ = 1.931 and γ_f_ = 2.207, the bubble is positioned far from the wall and at an intermediate distance from the free surface. In this case, the third impact value is relatively lower compared to the first and second impact values. In case II, where γ_w_ = 1.103 and γ_f_​=2.483, the bubble is closer to the wall, with γ_w_ falling within the humped range as discussed in Section 3.2. The characteristics of the signal indicate that the first impact has a relatively long duration (about 0.2 μs), while the subsequent impacts have shorter durations (about 0.1 μs). These durations will be used to calculate the impulse values later. Furthermore, the second maximum impact is greater than the first, and the time for the first impact is delayed relative to the others case I and III. A longer duration between the first and second peaks is observed compared to the other cases. Finally, in case III, where γ_w_ = 1.379 and γ_f_ = 1.379, the bubble is positioned at an intermediate distance from the wall and closer to the free surface. In this case, the impact signal is lower than in the others I and II.

**Fig. 5:**
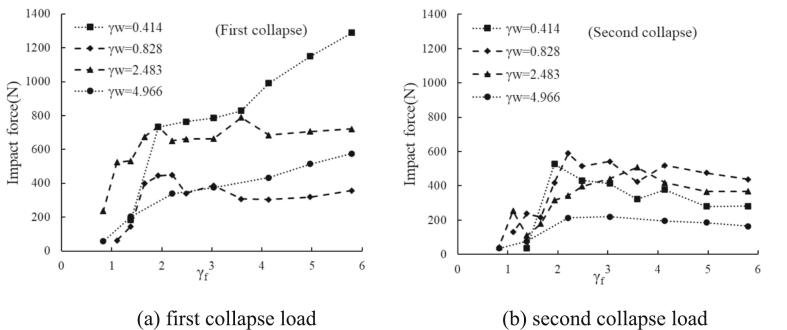
Comparisons for different γ_w_ as a function of γ_f._

The influence of the mixed boundaries on the bubble collapse characteristics is illustrated in Fig. 5. The comparisons of first and second loads for selected four γ_w_ s (0.414, 0.828, 2.483, and 4.966) are presented for γ_f_ < 6.0; see (a) and (b) of the figure respectively. The magnitude of the first loads decreases abruptly for γ_f_ < 2.0 for all γ_w_ cases; the slop is steepest for the closest distance to the wall (smallest γ_w_), while the intermediate distances have somewhat similar slop and the farthest one has a more gradual downward trend. For γ_f_ > 2.0, intermediate distances have almost plateau profiles, and the rest exhibit increasing trends. At γ_f_ ∼ 6.0, the most vigorous load is 1290 N (smallest γ_w_), which is followed by 720 N (γ_w_ = 2.483), 575 N (γ_w_ = 4.966), and 350 N (γ_w_ = 0.828). The lower impact loading features at γ_w_ ∼ 0.8 are the same as bubble collapse near a horizontal boundary under deep water reported in the preceding only solid wall section. Accordingly, the second impacts of this proximity parameter (γ_w_ ∼ 0.8) are higher than its first and second impacts of other parameter values, as evident in (b) of the Fig. 5. Comparable to the first load graph, second load curves for γ_f_ < 2.0 also fall noticeably, and the closer to the wall, the steeper the slope. The curves become more stable with deeper water depth.

**Fig. 6:**
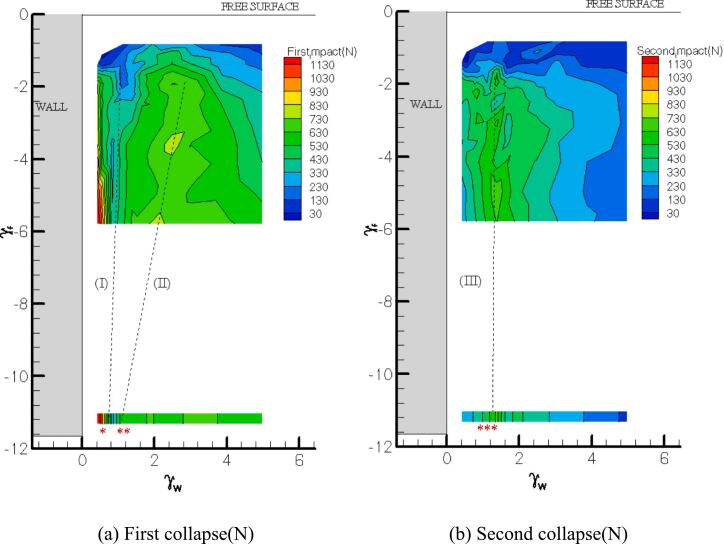

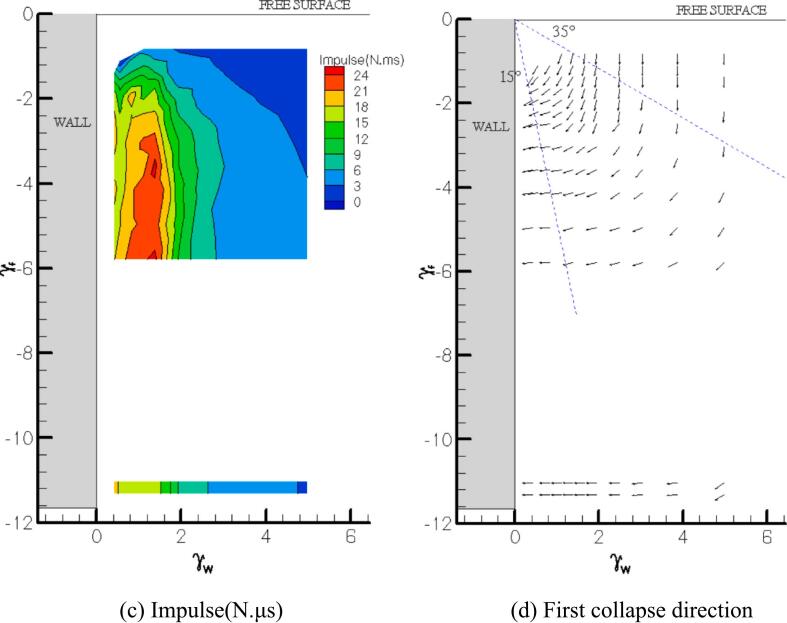
Color maps of (a) first collapse load, (b) second collapse load, (c) impulse, and (d) first collapse direction near free surface and a vertical wall.

Color contour maps of the first and second impacts near the free surface and a vertical wall are presented in Fig. 6(a) and (b) respectively. The numbers in the figure show maximum impact values from the experiments. The map includes over 8x7 explosion points (more points tested attempts at the corner). It is clear that γ_f_ < 2.0 indicates weak contour values for both first and second loadings. The linear dashed lines (I, II and III) are illustrated as pathway of minimum or maximum impact values. It is used for comparison with a single wall case (no free surface effect, section 3.2). The valley region (I) of the first collapse map in Fig. 6(a) slightly deviates from γ_f_ of 0.8 as the bubble moves closer to the water surface (The dash line I is continued to ‘*’ in Fig. 6(a)) indicating a position far from the free surface). It is unquestionably the lowest loading area (∼300 N) for the γ_w_ range employed in this study. The proximity of both the free surface and the solid wall introduces additional asymmetry, eliminating the formation of a compressed water layer. As a result, energy is predominantly released through the free surface. For stand-off distances γ_f_ > 2 (with respect to the free surface), a threshold appears to emerge, indicating the reappearance of compressed water layer effects. Consequently, the hump zone typically seen in the wall-only scenario does not develop near the free surface. Instead, the maximum impact load was recorded at approximately γ_w_ ∼ 2.7, indicating that the peak pressure occurs farther from the wall when a free surface is present. A more deviated pattern is seen for the recovery feature (II) when the bubble is created at a shorter distance from the free surface. The contour region becomes narrower with the smaller γ_f_. The dash line (II) connected to where almost no free surface effect (noted ‘**’ in Fig. 6(a)). In the second collapse contour map shown in Fig. 6(b), the highest second loads are acquired around γ_w_ ∼ 1.2 (III) and are more pronounced for γ_f_ > 3. Bubbles at deeper water depths deliver stronger second impacts(∼600 N) than at shallow water depth. The impulse values are also the largest at this similar distance from the boundary (∼24 N.μs), see Fig. 6(c). Bubble collapse loads near the boundary generally have fast-rising narrow base pulse signals, and those of far boundary are shock wave like signals. On the other hand, larger second collapse loads at region 'III' may add a substantial pulse to the signal. Thus, the impulse calculations of former profiles deliver smaller values than the latter (see Fig. 4(b)).

In Fig. 6(d), the first collapse direction is plotted to gain a rough impression of the combined boundaries’ influence on the bubble migration. Although jet orientation at the rebound stage changes its direction during re-expansion, it is found that the bubble ceroid motion is greatly related to the jet direction at first collapse. It is suggested that the free surface effect is stronger in the region 35° from the horizontal water line (almost vertical downward jetting), while the wall effect is prominent in the region 15° from the vertical line (almost horizontal jetting) for γ_f_ < 6.0 and γ_w_ < 5.0. Bubble dynamics are affected by both boundaries in the interval region. The 15° limit from the vertical wall may be underestimated for larger distances from the water line because wall influence becomes more and more noticeable for γ_f_ > 4.0. When both the free surface and the vertical wall are involved, the bubble collapse creates a jet that is directed from the free surface area towards the wall. The jet will generally follow an oblique or angled direction, starting from near the free surface and traveling downward toward the wall. This is due to the combined effects of the two boundaries. The free surface initially pushes the jet upward, but as the bubble moves farther from the surface and closer to the wall, the fluid is redirected towards the wall. The interaction between the fluid compression beneath the free surface and the wall's constraining effect leads to a jet path that generally points downward at an angle towards the wall. So, the presence of the wall plays a crucial role in shaping the direction of the jet. As the bubble is near the wall, the fluid cannot expand freely in all directions. Instead, the wall constrains the collapse and forces the jet to travel towards the wall.

## 5 Bubble under the grooved plate

**Fig. 7:**
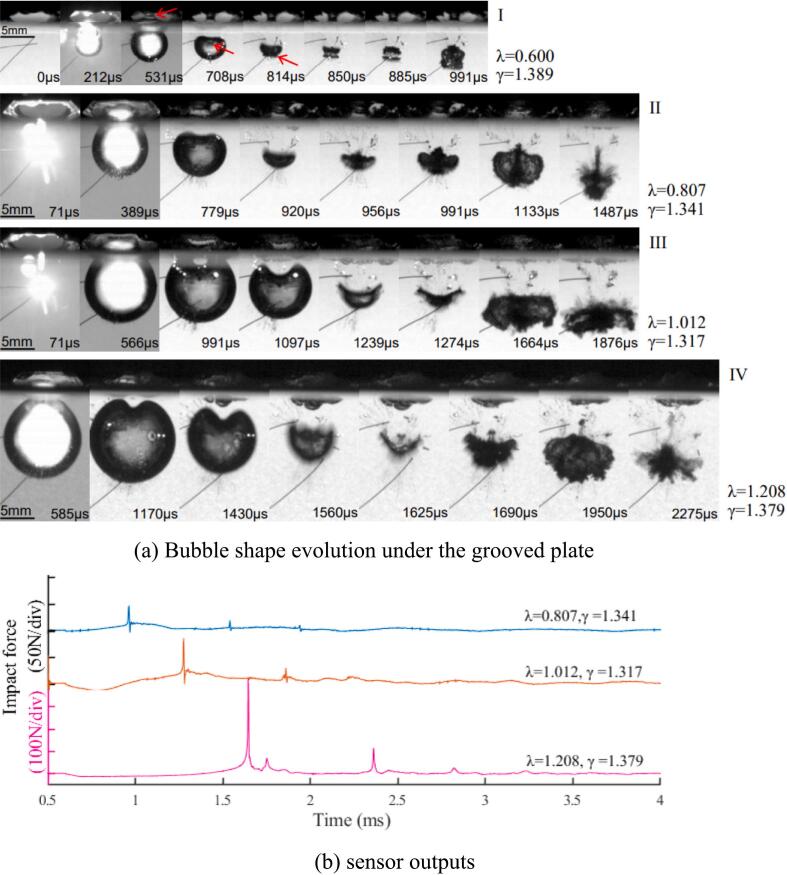
Bubble explosions beneath the confined air layer.

In the bubble explosion process beneath the free surface, especially during shrinking, the interface energy at the mesial side (the side facing the free surface) behaves in a way that significantly influences the behavior and energy transfer within the bubble. In this section, the bubble collapse under the grooved plate with or without an air pocket is investigated, the detailed sketch is described in section 2. Different collapse topologies are presented in Fig. 7(a), for similar stand-off parameters from the plate (γ ∼ 1.35). The ratio of the bubble diameter to the grooved length is λ; thus, four different bubble sizes are created at the same distance from the boundary. The smallest bubble (λ = 0.600) manifests the dome, spike, skirt formation and events of jetting, two toroidal collapses throughout its oscillation in a similar manner to the observation presented in the previous section. It implies that the confined free surface boundary is large compare with the bubble size and does not interfere with the bubble dynamic process at this certain birth point. Therefore, no signal is received by the sensor. In the λ = 0.807 case, the water dome formed by a growing cavity touches the boundary at the end of the bubble expansion, but no bursting occurs, and the bubble starts shrinking with a weak jet. In an open-air case, the sides of the dome fall back and facilitate the faster radial liquid flow in the axis of symmetry for an upward spike and downward jet. However, in this case, the liquid flow is greatly retarded at the left and right upper parts of the bubble because of the plate (groove). Therefore, inward flow to the shrinking bubble is more available from the direction perpendicular to the view, hence flow field is no longer radially symmetric near the boundary. This scenario is more evident in the larger λ because the groove interference becomes stronger, see III and IV of the figure. Consequently, the curvature at the bubble top surface is smaller with a larger bubble size. The collapsing bubble also pulls the free surface above it, forming an unstable surface. The bubble rebounds and migrates downwards eventually. The highest loading is 430 N by λ = 1.208, the second is 85 N by λ = 1.012 and the smallest is 47 N by λ = 0.807. Respective sensor outputs are given in Fig. 7(b).

**Fig. 8:**
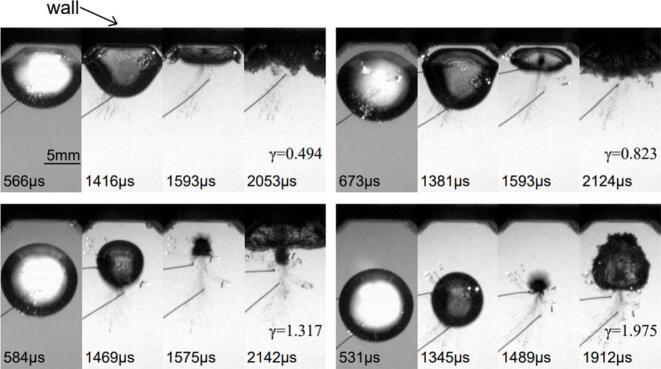
Single bubble collapse on the grooved plate as a function of γ.

**Fig. 9:**
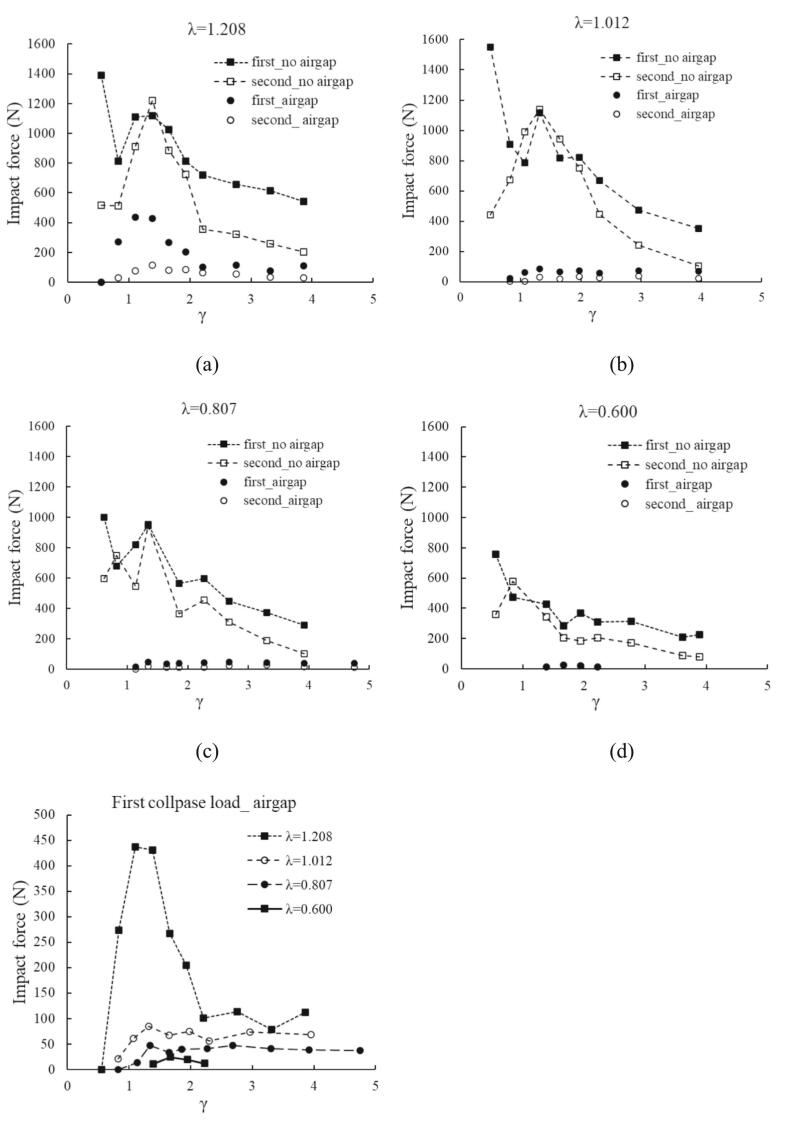
Bubble collapse impact under the plate with or without air gap for (a) λ = 1.208(R_max_ = 7.25 mm), (b) λ = 1.012(6.08 mm), (c) λ = 0.807(4.85 mm), (d) λ = 0.600(3.00 mm), and (e) first impact comparison with the air gap.

On the other hand, the experiments of bubble collapse under the plate without the air gap are also conducted for all four λ to clarify the effect of the air pocket. Fig. 8 depicts the typical bubble shape deformation near the plate. The bubble size shown in this case is 6.08 mm, which gives λ = 1.012. The difference between the flat boundary case and the patterned one is the presence of chamfer edges on the groove plate. These edges facilitate the wider bubble interface near the boundary during the growth phase and a longer bubble period, especially for smaller proximity parameters. After the first collapse, the rebound bubbles oscillate on the edges, and these impingements give strong loadings on the sensor readings. However, the collapse loads become significantly weaker for γ > 2.0, see Fig. 9(e).

The collapse loads with or without the air gap under the plate are compared in Fig. 9. It is confirmed that much stronger impingements on the boundary are detected without the air gap as expected. The first and second impact curves of λ > 1.0 are very similar to the general profiles; valley, recovery and second peak features. Except for the largest λ, the first and second loads with air gap are remarkably smaller than those of no air gap bubbles (approximately ten times) in Fig. 9(a)-(d). Especially, in Fig. 9(a), the biggest bubble with an air pocket hits the boundary with half the strength of no air gap case at the first collapse period, however second loads are much lower in contrast. Considering the sensing area of the PVDF sensor, the maximum value of λ was set to 1.208, and experiments were conducted at intervals of 0.2. The impact force at λ = 1.012 was approximately 70 % lower than that at λ = 1.208, clearly demonstrating the influence of the air layer on impact attenuation. In Fig. 9(e), the comparisons of first impact force under the air pocket are plotted. It is evident that the strength diminishes sharply for γ > 1.4 in all cases, and more steady curves are obtained for larger distances from the wall.

The presence of an air-water interface (here, air pocket) means that the collapse dynamics can be influenced by air entrainment which could absorb or dissipate part of the energy produced by the collapse. This interference significantly reduces the explosive potential of the bubble as it interacts with the air pocket formed at the surface.

## 6 Conclusion

The bubble is incepted near the combined boundary of the vertical rigid wall and the free surface.

The radius of the bubble applied to the experiment is a maximum of 7.25 mm, and the main event is completed within about 5 μs for a small bubble of this size. The bubble generator, PVDF sensor, and high-speed camera were synchronized, and the bubble's rebound event was successfully photographed. It causes the formation of water dome, spike, and skirt structures above the waterline when it is strongly affected by the free surface and weakly affected by the wall. Moreover, a comprehensive understanding of underwater explosion dynamics is achieved by the integration of the PVDF sensor. It is carefully treated by waterproof and protection layers to capture the very clear shockwave-like pulses generated by bubble implosions.

From the color impact load maps consisted by 36.25 mm × 43.5 mm plotted from the experiments, it is clear that γ_f_ < 2.0 indicates very weak contour values for both the first and second loadings because of energy dissipation to the free surface. In the contours, depending on the location of the bubble, the value of the impact force due to the first and second maximum collapses and the area of the valley-shaped impact force where occurring close to the wall are clearly visible. Compared to the case where there is no free surface effect, the second maximum collapses and valley regions can be said to be similar to the case of the vertical wall only, but the location of the first maximum collapses shows a bit large difference. This occurs about twice as far away as the vertical wall because of the free surface (if no free surface γ_w_ ∼ 1.4, with free surface γ_w_ ∼ 2.7). This dual-boundary configuration leads to sporadic dissipation of energy near the wall and free surface, which has minimal influence on the impact load near the boundary but contributes to increased impact pressure at intermediate distances. This effect is responsible for the observed doubling of impact load in certain regions.

The investigations of the air cushion effect under the grooved plate with the air gap are also conducted. By comparison, the impact loads without the air gap are much stronger than those of the plate with air gap. The existence of the water–air interface dramatically reduces the explosive level of bubble cavitation primarily due to energy dissipation and the interaction dynamics between the bubble and the free surface. Comparing the width of the groove with the diameter of the bubble(λ ratio), when the diameter of the bubble is smaller than the groove width, the maximum impact force is reduced by 1/10, and the greater the λ value, the greater the reduction. Even if the diameter of the bubble was larger than the groove width, the impact force was reduced by about 1/6. Therefore, it was quantitatively shown through the experiment that the impact force can be reduced when there is an air–gap between the bubble and the solid wall.

## CRediT authorship contribution statement

**Rho-Taek Jung:** Writing – review & editing, Writing – original draft, Visualization, Validation, Methodology, Investigation, Formal analysis, Data curation, Conceptualization. **NyoMeThetNaing:** Writing – review & editing, Writing – original draft, Visualization, Investigation. **Dong Kim:** Writing – review & editing, Writing – original draft, Validation, Supervision, Project administration, Methodology, Investigation, Funding acquisition, Formal analysis, Conceptualization.

## Declaration of competing interest

The authors declare the following financial interests/personal relationships which may be considered as potential competing interests: Dong Kim reports administrative support and article publishing charges were provided by National Research Foundation of Korea. Rho-Taek Jung reports administrative support and article publishing charges were provided by National Research Foundation of Korea. NyoMeThetNaing reports administrative support and article publishing charges were provided by National Research Foundation of Korea. The authors declare no conflict of interest. If there are other authors, they declare that they have no known competing financial interests or personal relationships that could have appeared to influence the work reported in this paper].
